# Physicochemical Properties of 4-(4-Hydroxyphenyl)-butan-2-one (“Raspberry Ketone”) Evaluated Using a Computational Chemistry Approach

**DOI:** 10.1021/acsomega.4c02293

**Published:** 2024-05-21

**Authors:** Peter A. C. McPherson, Niamh McKenna, Ben M. Johnston

**Affiliations:** † School of Pharmacy & Pharmaceutical Science, Ulster University, Coleraine BT52 1SA, U.K.; ‡ School of Pharmacy, University of North Carolina, Chapel Hill 27599, North Carolina, United States; § School of Science, Engineering & Construction, Belfast Metropolitan College, Belfast BT3 9DT, U.K.

## Abstract

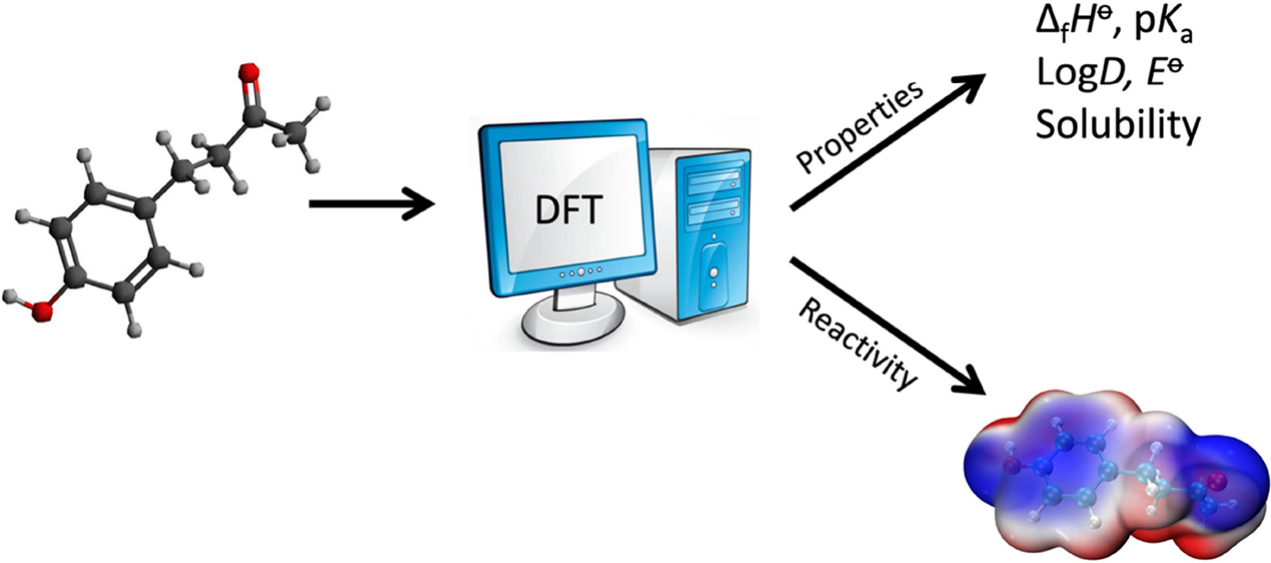

Raspberry ketone (RK) is a product of the phenylpropanoid pathway in a variety of plants and is the second most expensive natural flavouring in the world. It is also widely used as a nutritional supplement due to its reported ability to promote lipolysis and fat oxidation in vivo. We have evaluated the thermodynamics of RK using the correlation consistent ccCA-CBS-2 approach which afforded calculation of (inter alia) the enthalpy of formation. To obtain p*K*_a_, log *D*, electrode potential, solubility, and reactivity indices, we used TPSS/def2-TZVP geometries followed by single-point energies obtained at the M06-2X/def2-TZVPP level of theory. We obtained Δ_f_*H*^o^ = −299.4 ± 0.17 kJ·mol^–1^; the p*K*_a_ and log*D* were found to be 9.95 and 1.84, respectively, consistent with chemometric predictions. Using the enthalpy of fusion obtained from theory, we evaluated the aqueous solubility of RK to be in the region of 2.5 mg·mL^–1^ which is in agreement with limited literature reports. In terms of reactivity, we obtained a formal electrode potential of 1.29 V (vs SHE) at pH 7.4 and 298.15 K. The HOMO–LUMO energy separation in an aqueous environment was found to be ca. 7.8 eV, suggesting moderate chemical reactivity. Analysis of the frontier molecular orbitals using conceptual density functional theory supported this and revealed a reactivity pattern consistent with the metabolite profile obtained in mammals, namely, a propensity for nucleophilic attack at the carbonyl carbon and electrophilic addition of the benzene ring.

## Introduction

Raspberry ketone (RK), 4-(4-hydroxyphenyl)-butan-2-one, is a product of the phenylpropanoid pathway in various plants including raspberries (*Rubus idaeus* L.) where it serves as an attractant for pollinators and fruit flies.^1^ It was mainly used in the food and fragrance industry until it was popularized as having a positive effect on weight loss.^2^ Since then, it has been included in a range of nutraceuticals aimed at the weight loss market. Due to the low levels of RK present in plant sources, the majority of commercial RK is synthetic and can therefore only be marketed as a “nature identical” ingredient.^3^ Despite the widespread availability of RK, there exist little physicochemical or toxicological data, other than that obtained from chemometric methods. The lack of physicochemical data is likely an historical oversight due to the small quantities present in nature—RK was first identified in 1900 but attracted little attention outside of the perfume industry.^4^

The use of computational chemistry to determine physicochemical data is a well-developed field and is increasingly used in the drug discovery process to model key characteristics such as p*K*_a_ and log octanol partition coefficient (log *P*). Furthermore, with an increasing emphasis on sustainability and green chemistry, use of computational techniques rather than extensive laboratory trials has obvious appeal. Associated with this is the use of metrics to evaluate the sustainability of production methods which are based on the enthalpy of formation of the components.^5^ Where these data have not been determined experimentally, predictions based on group additivity methods are often used.^6^ However, computational chemistry can provide a more accurate prediction of these properties through ab initio and/or density functional theory-based methods.

Of the contemporary approaches available, the CCSD(T) method is considered the gold standard for theoretical thermochemistry, but it can be prohibitively expensive in terms of computational time even for medium-sized organic molecules.^7^ Alternatively, composite methods such as the Gaussian and Weizmann procedures are popular, mainly due to their ease of implementation in commercial software. A similar method is the correlation consistent composite approach (ccCA-CBS-2), which, unlike the Gaussian methods, does not depend on empirical parameterization.^8^ Instead, the ccCA-CBS-2 method uses a B3LYP/6-31G* optimized geometry to obtain MP2 energies with complete basis set extrapolation using the aug-cc-pV*n*Z (*n* = T, D, Q) basis set. This produces energies that are effectively the same as those that would be obtained using the QCISD(T) level of theory. Such composite methods are often used in conjunction with error-canceling schemes, such as isodesmic reactions which seek to balance the number and type of bonds on each side of a hypothetical equation.^9^ This approach is capable of agreement with experimental values to within 5 kJ/mol (often referred to as “chemical accuracy”).

Computational approaches have also been used to evaluate lipophilicity (as octanol partition coefficient)^10^ and p*K*_a_,^11^ both of which are crucial to understanding how a compound is absorbed and distributed in vivo. Insight into the metabolism and excretion of drug and drug-like compounds can similarly be gained through approaches such as conceptual density functional theory (CDFT),^12^ in which the overall reactivity of a molecule can be assessed through global reactivity parameters.^13^ Local reactivity indices such as those obtained from Fukui functions and average local ionization energy^14^ can then be used to predict the most likely sites of reactions that are characteristic of phase one biotransformation processes in vivo or that can be utilized in the synthesis of pro-drugs. This approach highlights the nexus of computational chemistry and quantitative structure–activity relationships whereby the former can be used to provide reliable data for predictive models.

We have undertaken an in silico analysis of raspberry ketone using density functional theory supported by a correlation consistent composite approach to obtain thermochemical values with an acceptable level of accuracy. Our investigation address a gap in the literature and provides an evaluation of the log octanol partition coefficient, p*K*_a_, aqueous solubility, electrode potential, and a conceptual DFT analysis, producing a detailed overview of raspberry ketone chemistry.

## Computational Methods

### General Approach

All structures were prepared using the molecule editor/visualizer program Avogadro,^15^ and electronic structure calculations were performed using Orca (Version 5.0.3).^16^ For all calculations except those used to determine Δ_f_*H*^o^, structures were initially optimized in the gas phase using the Tao–Perdew–Staroverov–Scuseria (TPSS)^17^ functional and def2-TZVP^18^ basis set with Grimme’s dispersion correction and Becke–Johnston damping. Single point energy calculations were then performed on the TPSS geometry at the M06-2X/def2-TZVPP level of theory.^19^ Gibbs energies were subsequently calculated using the total electronic energy obtained at the M06-2X/def2-TZVPP level with zero-point energy and thermal and entropic contributions derived from the TPSS/def2-TZVP frequency calculation. For modeling reactions in solvent phase, Truhlar’s universal solvation model density (SMD) was used as implemented in Orca.^20^ Optimized structures were confirmed through the absence of any imaginary modes.

### Thermochemistry

The Δ_f_*H*^o^ for RK was calculated using a range of isodesmic reactions (Table 1). Experimentally determined enthalpies of formation were obtained from the active thermochemical tables.^21^ The enthalpy of each species required for calculation of Δ_f_*H*^o^ was determined using the ccCA-CBS-2 method as implemented in Orca. This involves obtaining harmonic vibrational frequencies at the B3LYP/6-31G(*d*) level of theory followed by single point energies through the ccCA-CBS-2 extrapolation scheme. The final standard enthalpy (in kJ·mol^–1^) for each species is therefore obtained by
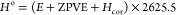
1where *E* is the ccCA-CBS-2 electronic energy; ZPVE is the zero-point vibrational energy and *H*_corr_ is the thermal enthalpy correction, both evaluated at the B3LYP/6-31G(*d*) level of theory. The final factor on the right-hand side is required to convert the units of energy from Hartree to kJ·mol^–1^. All thermodynamic terms were evaluated using the conventional rigid rotor harmonic oscillator (RRHO) approximation. The enthalpy of vaporisation (Δ*H*_vap_) and enthalpy of sublimation (Δ*H*_sub_) were determined according to the procedures outlined by Byrd and Rice^22^ using Multifwn to evaluate the molecular surface properties.^23,24^

**Table 1:** Isodesmic Reaction Schemes Used in the Evaluation of Δ_f_*H*^o^ for RK^a^

equation	reaction	Δ_r_*H*^o^	Δ_f_*H*^o^	*u*
(1)	C_10_H_12_O_2_ + CH_4_ ⇌ C_7_H_5_OH + CH_3_(CH_2_)_3_CO	11.54	–300.8	1.57
(2)	C_10_H_12_O_2_ + CH_4_ ⇌ C_6_H_5_OH + CH_3_(CH_2_)_4_CO	24.91	–302.8	1.33
(3)	C_10_H_12_O_2_ + CH_4_ ⇌ C_6_H_5_CH_3_ + CH_3_(CH_2_)_3_COOH	–52.12	–299.2	2.08
(4)	C_10_H_12_O_2_ + 2CH_4_ ⇌ C_6_H_5_CH_3_ + CH_3_(CH_2_)_3_CO + CH_3_OH	54.48	–294.7	1.22
(5)	C_10_H_12_O_2_ + CH_3_OH ⇌ C_6_H_5_OH + CH_3_(CH_2_)_3_COOH	–87.47	–295.9	0.99
(6)	C_10_H_12_O_2_ + CH_4_ ⇌ C_6_H_5_CH_2_OH + CH_3_(CH_2_)_3_CO	25.15	–302.7	1.99

a Values obtained from the ccCA-CBS-2 method. Units: kJ·mol^–1^_._

### Calculation of the Distribution Coefficient and Aqueous Solubility

The partitioning of a weakly acidic compound between polar and nonpolar environments at a specified pH can be described by the logarithmic distribution coefficient:^25^
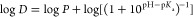
2

To calculate log *D*, we first evaluate the log octanol partition coefficient (log *P*) for RK using Gibbs energies that correspond to the RK(aq) ⇌ RK(octanol) equilibrium by way of the standard expression:
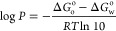
3

Next, the p*K*_a_ for RK was determined using an approach based on the inclusion of explicit water molecules.^26^ Gibbs energies were evaluated for each species in the acid–base equilibrium:
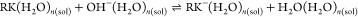
4where *n* is the number of explicit water molecules; in our approach, we took *n* = 2. The p*K*_a_ is then obtained by recognizing that eq 4 is composed of two separate equilibria and will therefore be a function of their equilibrium constants:
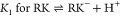
5a
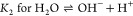
5b

This gives *K*_a_ = *K*_1_*K*_2_ and taking *K*_2_ = 1 × 10^–14^ and [H_2_O] = 55.33 M, we obtain the following expression (after taking logarithms):
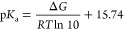
6where the constant on the right-hand side of the expression is the p*K*_a_ of water.^27^ The value of log *P* can also be used to predict the aqueous solubility of a crystalline solid such as RK using the general solubility equation:^28^
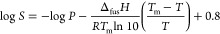
7where log *S* is the logarithm of the molar solubility, Δ_fus_*H* is the molar enthalpy of fusion, *T*_m_ is the melting point (357.58 K), and *T* is the temperature at which solubility will occur (298.15 K). The constant on the right-hand side is empirically derived and includes a factor to convert logarithmic mole fraction solubility to the more customary log *S*.

### Electrode Potential

**Figure 1:**
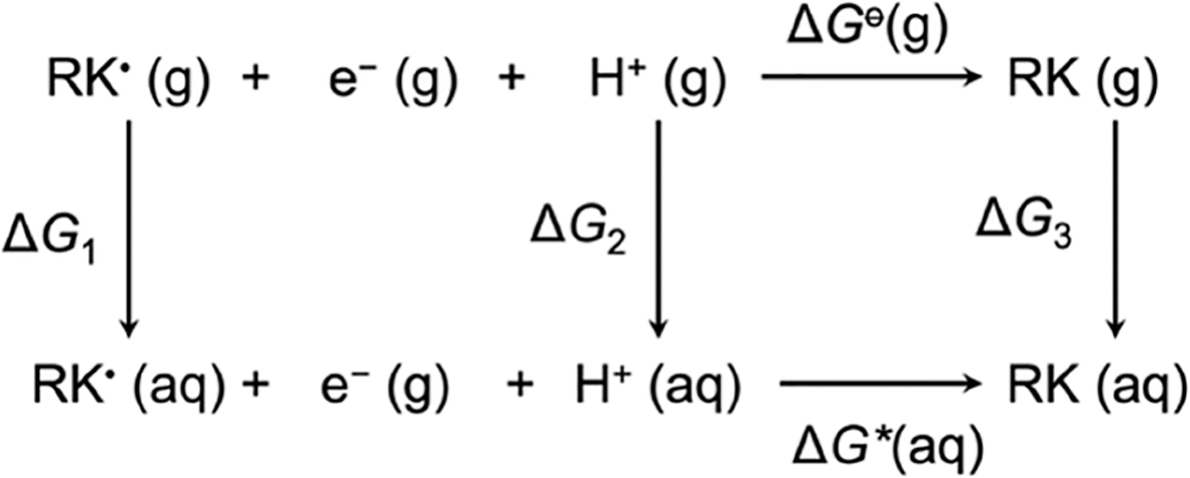
Thermochemical cycle for determination of Δ*G*_aq_^*^ and the associated electrode potential.

Under conditions where RK is fully disassociated to the corresponding phenolate anion, the oxidation would proceed in a straightforward one-electron process. However, as RK is largely un-ionized when pH < 10, the oxidation will involve the loss of a proton and therefore become a pH-dependent process. The absolute electrode potential, *E*, for RK can be calculated using a thermochemical cycle^29^ (Figure 1) which yields a value for Δ*G*_aq_^*^. This is then used to determine *E* via the Nernst equation:
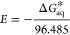
8where the factor in the denominator (the Faraday constant) is required to convert units of energy from kJ·mol^–1^ to volts. Note that the superscript asterisk denotes a 1 M reference state, i.e., Δ*G*_aq_^*^ = Δ*G*_aq_^o^ + *RT* ln (24.46). The electrode potential is then expressed relative to the standard hydrogen electrode, taking *E* (SHE) = 4.44 V.^30^

### Global and Local Reactivity

The chemical reactivity of RK can be predicted to some extent through the use of global and local reactivity parameters. The first of these, the electronic chemical potential (μ), can be broadly considered as the ability of a molecule in its ground state to exchange electron density with its environment, and it is approximated by the expression:
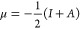
9where *I* is the vertical ionization energy and *A* is the electron affinity of the molecule, although Koopmans’ approximation is usually invoked such that *I* ≈ −*E*_HOMO_ and *A* ≈ −*E*_LUMO_.^31^ The opposite case, the resistance of a molecule to an exchange of electron density with its environment, is regarded as its chemical hardness (η), and this is usually taken as
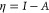
10

Note that in eq 10, we do not take half the difference in energy as is often reported in the literature; see Pearson.^32^ The regions of a molecule that are likely to undergo reaction with nucleophiles, electrophiles, and free radicals can be predicted by the molecular electrostatic potential and average local ionization energy.^33^ These are computed on the surface of the molecule, where local minima reveal the location of the least tightly held electrons (for average local ionization energy) or most negative electrostatic potentials (for molecular electrostatic potential). Determination of all reactivity descriptors was performed using Multiwfn^34^ using energies obtained at the M06-2X/def2-TZVPP level of theory. Topological isosurface plots were produced using VMD.^35^

## Results and Discussion

### Thermodynamic Properties

The standard molar enthalpy of formation for RK was calculated as −299.4 ± 0.17 kJ·mol^–1^ which is the average ±95% confidence interval of the values shown in Table 1. This value is consistent with that obtained from group contribution methods (−303.09 kJ·mol^–1^) and can reasonably be assumed to be within ±5 kJ·mol^–1^ of the true value due to the extensive benchmarking of the ccCA-CBS-2 method. The heat capacities and molar entropy were computed directly using the RRHO approximation as *C*_v_ = 187.4 4 J·K^–1^·mol^–1^, *C*_p_ = 195.75 J·K^–1^·mol^–1^, and *S*^o^ = 477.11 J·K^–1^·mol^–1^, which in turn gives Δ_f_*G*^o^ = −441.65 kJ·mol^–1^.

Although Δ_f_*H*^o^ can be computed from reaction enthalpies of combustion, we opted to use isodesmic reaction schemes (where the numbers and types of bond present in products and reactants are the same) due to the favorable cancellation of errors.^36^ Of course, the enthalpy of formation for RK could be determined experimentally from bomb calorimetry data. However, as this is usually conducted with samples in the condensed state, the enthalpy of vaporization or sublimation would be required, necessitating further experimental measurements with their respective errors. Byrd and Rice demonstrated that Δ_vap_*H*^o^ and Δ_sub_*H*^o^ could be determined theoretically from three molecular surface properties, viz., the surface area of the electron density of the molecule, the variation in the surface electronic potential, and the balance of the positive and negative surface charges.^37^ This method outperforms models based on group additivity/quantitative structure–activity relationships and when combined with data obtained for Δ_f_*H*^o^ in the gas phase permits estimation of Δ_f_*H*^o^ in the condensed state via Hess’s law. We obtained Δ_vap_*H*^o^ = 70.03 and Δ_sub_*H*^o^ = 96.95 kJ·mol^–1^.

### Geometry Optimization for Physicochemical Properties

**Figure 2:**
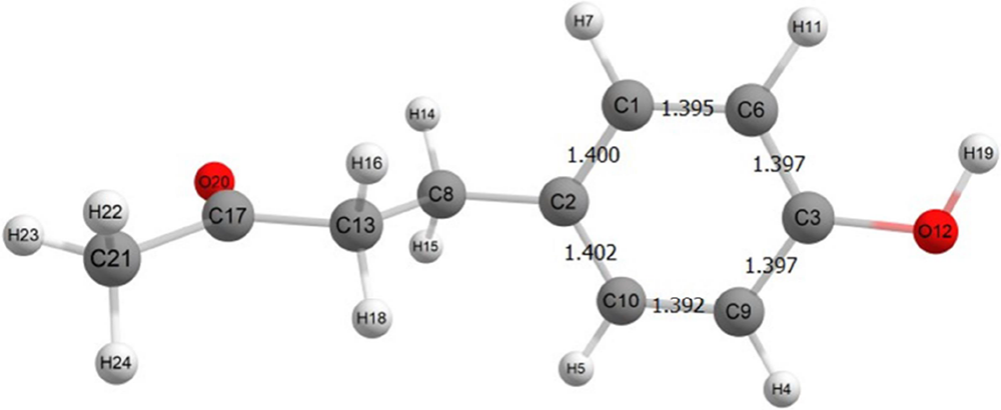
TPSS/def2-TZVP optimized geometry and atomic numbering scheme. Bond lengths are given in angstrom.

The optimized geometry for RK was first established in the gas phase using the meta-GAA TPSS functional with the def2-TZVP basis set based on the findings of Isegawa et al.^38^ This functional compares well to the more common B3LYP functional but typically has a shorter computational time. Inspecting the structure of RK, we see that it has one freely rotatable bond on the butanoyl substituent (C_2_–C_8_; see the numbering scheme in Figure 2) that could give rise to low-energy conformers. To investigate this, a scan of the dihedral angle (θ) formed by the atoms C_2_–C_8_–C_13_–H_18_ was performed in the range 180° ≤ θ ≤ 360°. This revealed three conformers with the lowest energy structure being separated from the other two by ca. 5 kJ·mol^–1^. The most stable conformer is asymmetrical with respect to the phenyl ring which is inclined at an angle of 75.9° from the planar butan-2-one substituent. The dihedral angle θ = 55.1° is close to that found in the crystal structure (θ = 51.8°),^39^ and in general, all bond lengths/angles are in good agreement with these experimental measurements (see Tables S1 and S2 in the Supporting Information). Although hybrid density functionals are known to exhibit marginal spin contamination, in all our calculations the average deviation of ⟨S^2^⟩ values from the eigenvalue *S*(*S* + 1) = 0.75 do not exceed 2%, so overall the TPSS/def2-TZVP optimization seems adequate for our purposes.

### Distribution Coefficient and Aqueous Solubility

The partition coefficient is a key metric used to evaluate the behavior of drugs and drug-like compounds in vivo. It is often included in empirical “rules of thumb” such as Lipinski’s rule of five which states that molecules with 1.35 ≤ log *P ≤* 1.80 should have good oral bioavailability.^40^ Using the TPSS/def2-TZVP//M06-2x/def2-TZVPP scheme, we obtained log *P* = 1.83 which is very similar to the values obtained from chemometric methods, e.g., SwisADME returns log *P* = 1.84.^41^ However, as RK has an ionizable group, the p*K*_a_ should be taken into account when considering oral bioavailability, which can be achieved via the log distribution coefficient (log *D*).

To obtain log *D*, we first calculated p*K*_a_ = 9.95 which is slightly lower than that predicted using chemometric methods (p*K*_a_ = 9.99) but is consistent with a phenol bearing an electron-withdrawing group (i.e., the p*K*_a_ is expected to be lower than phenol which has p*K*_a_ = 10). In the absence of a reliable experimental value, we can only judge the accuracy of our prediction based on the known limitations of p*K*_a_ determination by computational techniques. That said, experimental values themselves for p*K*_a_ typically incur errors in the region of 0.01–0.10 p*K*_a_ units which gives some tolerance for comparison to theoretical values. Direct approaches for calculation of p*K*_a_ require knowledge of the free energy of solvation for a proton which has a range of values in the literature.^42^ We have overcome the need for this value by calculating p*K*_a_ using a neutralization reaction in a cluster-continuum solvation model. A crucial aspect of this approach is the orientation and position of explicit water molecules so that the short-range solute–solvent interactions (chiefly hydrogen bonds) are adequately modeled. In the present study, we placed two water molecules adjacent to the hydroxyl group undergoing protonation/deprotonation which has previously been shown to be effective for modeling the p*K*_a_ of phenols.^43^ Overall, the impact of any uncertainty in the prediction of the p*K*_a_ will have minimal impact on the calculation of log *D* as log *P* is the major contributor for this function. We found that log *D* = 1.83 which places RK on the upper boundary of what is considered to have good oral bioavailability. Only in situations where pH ≥ p*K*_a_ will log *D* decrease by any appreciable amount (e.g., at pH = p*K*_a_, log *D* = 1.53), and obviously, such conditions will not arise in vivo.

A final major factor in determining oral bioavailability is solubility in water. As is the case with other properties, reliable experimental values for the aqueous solubility for RK are scarce. One study employing gravimetric measurements reports the solubility as 0.05 M at 298 K,^44^ while chemometric predictions range from 0.001 to 0.014 M (0.16–2.30 mg·mL^–1^) which would place RK within the very slightly soluble to slightly soluble range of the European Pharmacopeia. We evaluated solubility using a general solubility equation based on that originally developed by Ran and Yalkowsky.^45^ This approach requires knowledge of the melting point and enthalpy of fusion; we estimated the latter from Δ_vap_*H*^o^ and Δ_sub_*H*^o^ obtained from the ccCA-CBS-2 thermochemistry. This gave Δ_fus_*H*^o^ = 26.92 kJ·mol^–1^ which compares favorably with the literature value 22.75 kJ·mol^–1^.^46^ Taking our value for Δ_fus_*H*^o^, we obtained a molar solubility of 0.0153 M (2.51 mg·mL^–1^); for comparison, using the literature value for Δ_fus_*H*^o^ returns a solubility of 0.0202 M (3.32 mg·mL^–1^). In subsequent work, eq 8 was simplified to employ Walden’s rule which sets Δ_fus_*S* = 56.5 J·K^–1^·mol^–1^, and thus, only the melting point is required for estimation of solubility. This approach would yield a solubility of 0.012 M (1.97 mg·mL^–1^).

### Electrode Potential

The electrode potential for the RK radical/RK couple vs SHE was found to be *E*^o^ = −0.85 V (Δ*G*_aq_^*^ = −510.48 kJ·mol^–1^). However, as this redox process involves proton transfer, the value is pH-dependent and can be adjusted to the corresponding formal potential by
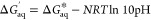
11where *N* is the number of hydrogen atoms transferred. This returned *E*′ = 1.29 V (Δ*G*_aq_^′^ = −552.7 kJ·mol^–1^) at pH 7.4 and 298.15 K. The significance of the electrode potential is that many plant-derived phenols are considered to be antioxidant which can be crudely judged by its electrode potential. For example, thymol has *E*′= 1.04 V (vs SHE) at pH 7.4, which is sufficient to bring about reduction of many oxidized biomolecules such as lipid hydroperoxides.^47^ Given that RK would be considered as amphiphilic based on its log*P*, it is reasonable to expect the molecule to freely diffuse throughout all cellular environments (membrane and cytosol) and could therefore participate in regeneration of endogenous antioxidants such as α-tocopherol (TOC), i.e., TOC^•^ + RK ⇌ TOC + RK^•^, Δ*G*′ = −176.3 kJ·mol^–1^. The caveat for this reaction is that the RK radical may undergo reversible transformation to a quinone, a species which is known to be damaging to the cellular environment.

### Chemical Reactivity

The global reactivity of a molecule such as RK can be described by a range of properties that originate from conceptual density functional theory. If we consider a chemical reaction in terms of the frontier molecular orbitals, we expect the LUMO of the electrophile to act as an electron acceptor, while the HOMO of the nucleophile serves as an electron donor. It follows that there is often (but not always) a correlation between the HOMO–LUMO energy separation and chemical reactivity.^48^ In a bimolecular reaction, the smaller the energy separation, the greater the interaction between the two frontier orbitals which has a stabilizing effect on the transition state. A quantitative outworking of this theory is the Mayr equation which relates the rate of reaction with the respective electrophilicity or nucleophilicity of the species involved.^49^

For RK, the energies of frontier orbitals are shown in Table 2 which gives rise to a HOMO–LUMO energy gap (Δ*E*) of around 7.8 eV. This would suggest that RK is moderately stable; for comparison, phenol has Δ*E* = 8.2 eV, yet it is known to readily undergo electrophilic substitution of the phenyl ring. This discrepancy may be a result of direct evaluation of Δ*E* as the difference in HOMO and LUMO eigenvalues rather than through the use of time-dependent density functional theory (TD-DFT).^50^ This alternative approach is given in Figure S1 (Supporting Information). The electronic chemical potential, or the ability of a species to exchange electron density with its environment, can be regarded as the negative of the absolute (Mulliken scale) electronegativity, i.e., −μ = χ, and hence, we see that RK is likely a modest electron-accepting species. The companion measurement, chemical hardness, helps further characterize this nature through HSAB (hard–soft acid–base) theory, and from the values in Table 2, we conclude that RK is a relatively hard Lewis acid (large Δ*E,* low η) and would therefore be expected to react with a hard Lewis base (a species that is weakly polarizable with a high-energy HOMO). The variation in ionization potential and electron affinity across the three environments is a reflection of solute–solvent interactions; for example, it has previously been established that the electron affinity is greater in solvents with large dielectric constants.^51^ Electron affinity is similarly affected by the geometry relaxation of the anions in different solvents.^52^ The electrophilicity index, ω = μ^2^/2η, can be regarded as a summary of these previous measures, and we see that in a polar environment, RK has marginally enhanced electrophilicity over the nonpolar environment. The reactivity of RK is further supported by a modest dipole moment (4.5 D) which is significantly higher than that of phenol (1.32 D) or butanone (3.20 D), which supports the overall view that RK should be sufficiently reactive toward biotransformation reactions in vivo.

**Table 2:** CDFT Properties of RK^a^

	gas	aqueous	lipid^b^
ionization potential	8.364	6.191	6.734
electron affinity	–1.139	0.884	0.402
*E*_HOMO_	–7.53	–7.51	–7.52
*E*_LUMO_	0.31	0.35	0.33
electronic chemical potential	–3.61 (−3.61)	–3.58 (−3.55)	–3.59 (−3.57)
chemical hardness	7.84 (4.75)	7.86 (2.65)	7.84 (3.17)
electrophilicity index	1.66 (1.37)	1.63 (2.36)	1.65 (2.01)

a Values in parentheses are computed from the vertical ionisation potential and electron affinity. All values in eV.

b Computed using pentyl acetate to mimic the lipid environment.

As Koopmans’ approximation is known to be dependent on the functional used,^53^ we sought to verify the conclusions made from FMO evidence by computing the vertical ionization potential and electron affinity for RK in each environment. This was achieved by taking the TPSS/def2-TZVP ground-state geometry and determining the energies of the ionic states, *E*(M^+^) and *E*(M^–^), at the M06-2X/def2-TZVPP level. Thus, the ionization potential and electron affinity are obtained relative to the bottom of the ground-state potential well as IP = *E*(M^+^) – *E*(M) and EA = *E*(M) – *E*(M^–^). We recalculated μ, η, and ω using these values (Table 2, values in parentheses) and found that while the overall trend was similar, use of Koopmans’ approximation appeared to overestimate the respective indices. This is consistent with the view that long-range corrected functionals satisfy Koopmans’ approximation as they are capable of reproducing the orbital energies in a more complete way.

**Figure 3:**
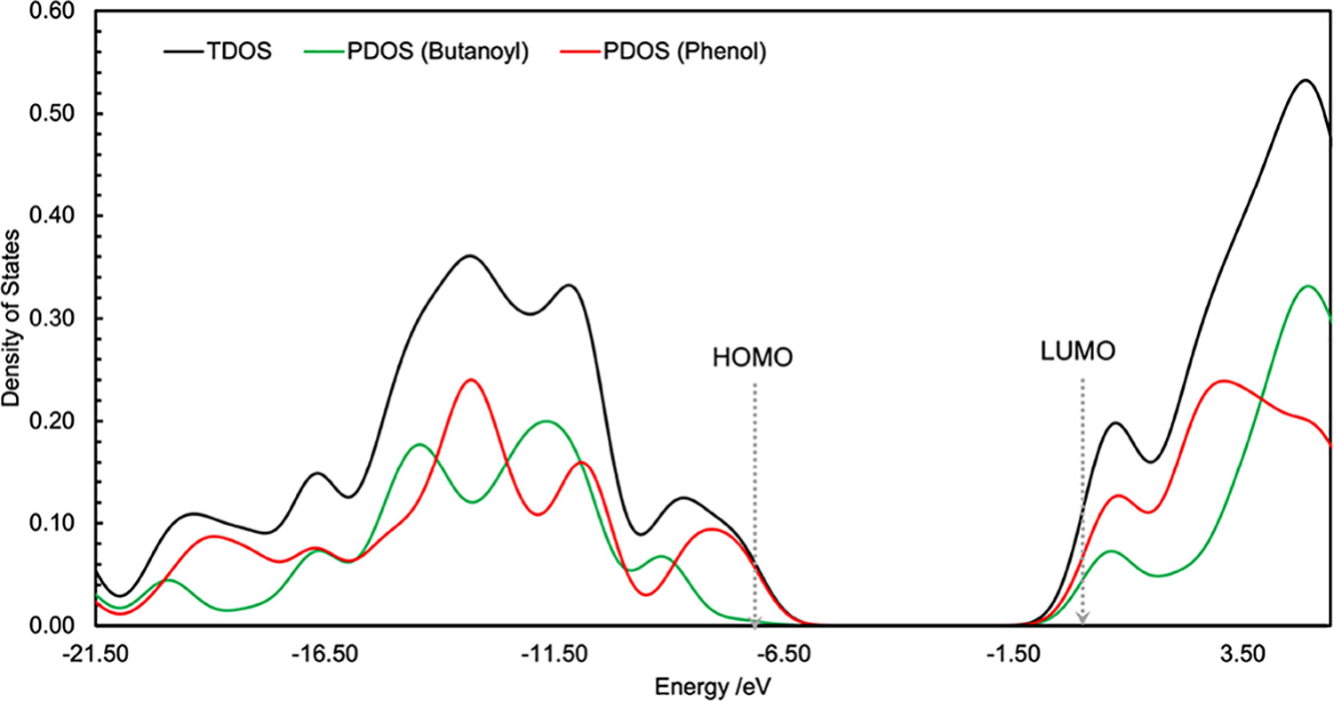
Density of states analysis for RK. Abbreviations: TDOS, total density of states; PDOS, partial density of states.

To complete our global investigation of RK’s reactivity, we performed a density of states analysis (Figure 3) in which the total density of states (TDOS) represents the proportion of states occupied by the system at each energy level. This can be decomposed into a partial density of states (PDOS) by splitting the RK molecule into two main fragments, the butanoyl substituent and the phenol group, and plotting their respective densities. From this, we see that the phenol substituent is the major contributor to the HOMO density (Figure 3, peak on red line at ca. −7 eV) while the LUMO appears to be relatively delocalized over both fragments (i.e., both are making contributions to the LUMO density; Figure 3, red and green lines at ca. 0.3 eV). Of course, while it is tempting to align a molecule’s reactivity with the location of FMOs, other considerations should be taken into account, such as loss of resonance stabilization on addition of a species to an aromatic system.^54^ Likewise, since molecular orbitals will inevitably become distorted along the reaction coordinate, a more comprehensive kinetic analysis is often required.

**Figure 4:**
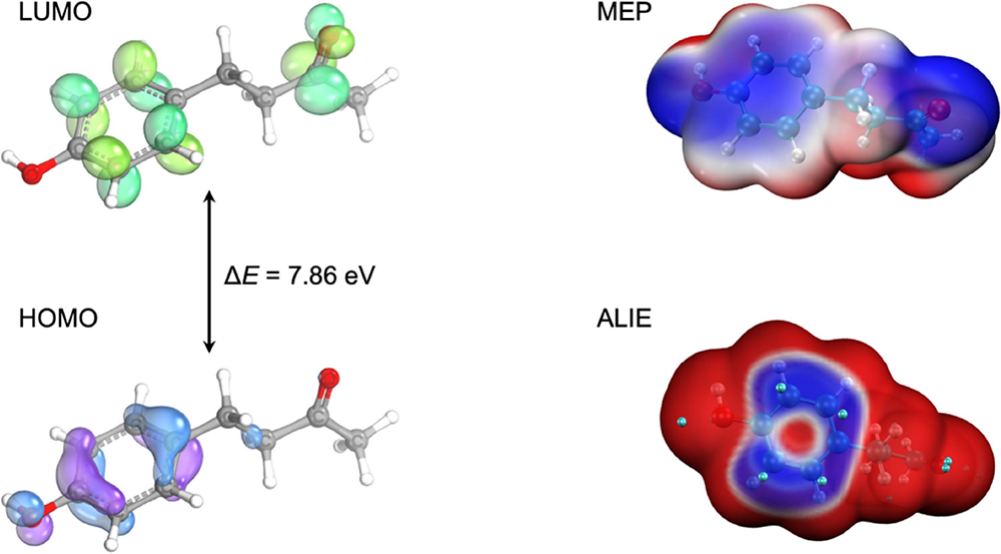
(A) Representations of LUMO and HOMO. (B) Molecular electrostatic potential (blue isosurface represents regions of high electrostatic potential, isosurface value 0.001) and average local ionization energy (blue isosurface represents regions of low ionization energy, isosurface value 0.0005).

We see an alternative view of this reactivity pattern in Figure 4 where the larger lobe of the LUMO is centered over C17 (the carbonyl carbon) which is supported by the topological plot of the molecular electrostatic potential (MEP). The blue regions of the MEP isosurface indicate regions of higher negative electrostatic potential. This view supports in vivo findings^55^ in which the major metabolite of RK is formed through reduction of the carbonyl to a secondary alcohol, forming 4-[(3*R*)-3-hydroxybutyl]phenol (rhododendrol). When we examine the HOMO plot, we see that the larger lobes are centered over C1, C6, C9, and C10, which is supported by the average local ionization energy (ALIE) isosurface plot. In the latter, blue regions are those with a low ionization potential and therefore likely sites for electrophilic additions. In fact, if we compare these findings to Figure 2, we see that the bond lengths between C1–C6 and C9–C10 are shorter than those elsewhere in the phenyl group, suggesting that the former have more double bond character. Again, comparison with metabolites found in animal models shows formation of a C6-hydroxy metabolite [4-(3,4-dihydroxypenyl)butan-2-one], which is consistent with the theoretical reactivity pattern established in our study.

## Conclusions

Density functional theory can be a powerful predictive tool that is a good compromise between speed and accuracy. It allows in silico evaluation of properties of relevance to drug design in a way that reduces dependence on extensive empirical data (unlike QSAR). Using raspberry ketone as an example for which there exist little physiochemical data, we have computed a range of properties that have their origins in the thermodynamics of the molecule. The enthalpy of formation was found to be −299.4 ± 0.17 kJ·mol^–1^ using the extensively benchmarked ccCA-CBS-2 approach. Evaluation of the p*K*_a_ and log *D* yielded values of 9.95 and 1.84, respectively, consistent with chemometric predictions. For the aqueous solubility, we obtained a value of 0.015 M which is in agreement with other predicted values. The redox activity of RK was confirmed by calculation of its formal electrode potential (1.29 V vs SHE at pH 7.4 and 298.15 K), which implies a potential for modest antioxidant functions. The broader reactivity of RK was determined through CDFT through which we found that the phenyl ring can be expected to undergo electrophilic additions due to a greater low local average ionization potential. On the other hand, the butanoyl chain has a more negative electrostatic potential, which supports reduction of the carbonyl group to an alcohol. These latter findings are supported by studies of metabolites formed in animal models and, more broadly, are consistent with well-established reactivity trends for functional groups.

## Supporting Information Available

Comparison of experimental geometry (bond lengths and dihedral angles) vs TPSS/def2-TZVP optimized structure and theoretical and experimental ultraviolet spectrum (PDF)
