# Supplementary material for: Physicochemical Properties of 4-(4-Hydroxyphenyl)-butan-2-one (“Raspberry Ketone”) Evaluated Using a Computational Chemistry Approach
Source: ACS Omega. 2024 May 21;9(22):23963–70. doi: 10.1021/acsomega.4c02293 (PMC11154730; doi:10.1021/acsomega.4c02293)

Physicochemical Properties of 4-(4-Hydroxyphenyl)-butan-2-one (‘Raspberry Ketone’) Evaluated Using a Computational Chemistry Approach

Supporting Information

Peter A C McPherson*[1], Niamh McKenna[2] and Ben M Johnston[3]

[1]School of Pharmacy & Pharmaceutical Science, Ulster University, Coleraine, UK; [2]School of Pharmacy, University of North Carolina, Chapel Hill, USA; [3]School of Science, Engineering & Construction, Belfast Metropolitan College, Belfast, UK.

Table S1 Bond lengths (XRD *vs*. TPSS geometry). Refer to numbering scheme in Figure 2.

Atoms	XRD	DFT	Atoms	XRD	DFT
C3-C6	1.371	1.397	C8-C13	1.525	1.537
C3-O20	1.372	1.376	C8-H14	0.970	1.095
C3-C9	1.384	1.397	C8-H15	0.970	1.095
C6-C1	1.384	1.392	C13-C17	1.501	1.525
C6-H11	0.930	1.086	C13-H16	0.970	1.100
C1-C2	1.379	1.402	C13-H17	0.970	1.100
C1-H7	0.930	1.088	C17-O20	1.200	1.219
C2-C10	1.386	1.400	C17-C21	1.489	1.520
C2-C8	1.508	1.513	C21-H22	0.960	1.096
C10-C9	1.382	1.395	C21-H23	0.960	1.096
C10-H5	0.930	1.088	C21-H24	0.960	1.096
C9-H4	0.930	1.089	O12-H19	0.910	0.971

Table S2 Dihedral angles (XRD *vs*. TPSS geometry). Refer to numbering scheme in Figure 2.

Atoms	XRD	DFT	Atoms	XRD	DFT
O1-C1-C2-C3	−178.8	−179.8	O1-C1-C6-C5	179.6	179.9
C6-C1-C2-C3	−0.3	−0.2	C3-C4-C7-C8	−102.6	−91.3
C1-C2-C3-C4	−0.7	−0.1	C5-C4-C7-C8	75.6	87.4
C2-C3-C4-C5	0.9	0.3	C4-C7-C8-C9	−173.6	−179.1
C2-C3-C4-C7	179.1	178.5	C7-C8-C9-O2	-3.5	4.1
C3-C4-C5-C6	0.0	0.3	C7-C8-C9-C10	176.7	175.7
C7-C4-C5-C6	−178.3	−178.5	C4-C5-C6-C1	−1.0	−0.6
C2-C1-C6-C5	1.2	0.2			

Experimental data from: C Wang, J. G. (2011). 4-(4-Hydroxyphenyl) butan-2-one. *Acta Crystallographica Section E: Structure Reports Online*, *67*(6), o1411-o1411. https://doi.org/10.1107/S1600536811017272

Figure S1 Theoretical ultraviolet absorption spectrum (solid thick line) for RK obtained at the TPSS/def2-TZVP level using time dependent density functional theory (TD-DFT) and ESD (excited states dynamics) using ethanol as a model solvent. The total ultraviolet spectrum was decomposed into the major transitions: S0 → S2 (—); S0 → S3 (– – –); S0 → S4 (----) and S0 → S5 (·····). From the ESD output, we identified the S0 → S2 transition as the major contributor (*ca*. 69%) to the HOMO−LUMO band gap. For reference, the experimental spectrum (inset) has a peak in this region at 275 nm (corresponding to 4.5 eV). The difference in absorption (experimental *vs*. theoretical) is due to vibronic coupling.

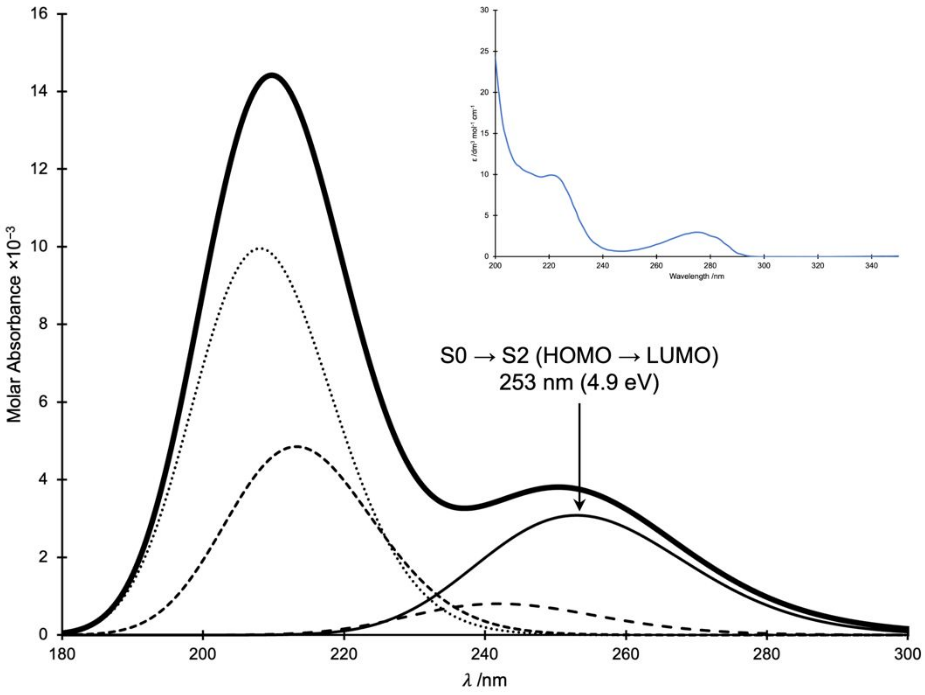

Supplement: Supplementary file 1 — ao4c02293_si_001.pdf [file ao4c02293_si_001.pdf]
